# Gaze and gait dynamics are shaped by terrain type during urban walking

**DOI:** 10.1038/s41598-026-57119-8

**Published:** 2026-06-17

**Authors:** Simon Ladouce, C. R. Gillebert

**Affiliations:** 1 Brain and Cognition, Leuven Brain Institute, KU Leuven, Leuven, Belgium; 2 Department of Architecture, University of Cambridge, Cambridge, United Kingdom

**Keywords:** Walking, Locomotion, Gait, Gaze, Eye-tracking, Attention, Terrain, Urban environment, Spatial analysis, Ecological validity, Engineering, Neuroscience

## Abstract

The structure of the environment fundamentally shapes how humans see and move. Walking through the city requires the continuous coordination of eyes, head, and body movements, revealing the active role of perception in guiding locomotion. Here, we used wearable eye-tracking, foot-mounted inertial sensors, and GPS to examine how terrain type modulates gaze behaviour and locomotor dynamics during naturalistic urban walking. Twenty young adults walked along routes comprising flat pavements, cobblestones, and dirt paths while their gaze, head orientation, and gait were recorded. Gaze behaviour varied systematically with surface irregularity: cobblestones and dirt paths elicited more frequent downward, proximal gaze and larger downward head-pitch deviations. Gait also adapted, with slower pace, shorter strides, and reduced cadence on uneven surfaces. Spatial correlations revealed tightly coupled adjustments between gaze and gait, indicating continuous sensorimotor coordination. These findings show how the physical structure of urban environments shapes predictive visuomotor strategies during real-world locomotion, providing a multimodal foundation for understanding embodied cognition in motion.

**Supplementary Information:**

The online version contains supplementary material available at https://doi.org/10.1038/s41598-026-57119-8.

## Introduction

Walking through a city is one of the most habitual human activities. While it may feel effortless (both physically and cognitively) for most healthy individuals, this apparent ease reflects the outcome of lifelong-acquired expertise^1, 2^. At its core, walking relies on a continuous interplay of perceptual, motor, and cognitive processes that enable stability, obstacle avoidance, and forward planning. For much of daily urban life, these mechanisms operate beneath awareness as people traverse flat, predictable pavements. Yet their hidden complexity becomes apparent when the ground surface changes, such as when stepping onto cobblestones or dirt paths scattered with rocks and irregularities, or when conditions such as fatigue, injury, or motor impairment challenge the body’s ability to sustain smooth and automatic coordination. As cities are projected to host over 60% of the global population by 2030 (United Nations^3^), understanding how individuals adapt their walking behaviour to diverse urban environments is increasingly relevant for both science and society. On average, people living in cities take around 5,500 steps per day^4^, corresponding to nearly an hour of walking^5^.

These seemingly automatic behaviours therefore unfold through a finely tuned orchestration of overt visuospatial attention and motor control, which together form an integrated visuomotor system (the coordinated coupling between visual and motor processes that supports adaptive movement) that allows individuals to navigate terrains ranging from smooth pavements to uneven cobblestones and dirt paths. Throughout this paper, visuomotor behaviour refers to this coupling between gaze behaviour and locomotor control during walking. In this study, we use gaze direction as an observable proxy for the visual information being sampled from the environment and discuss overt visuospatial attention as an interpretation of these gaze behaviours.

Understanding these adaptive visuomotor behaviours requires considering perception and action as components of an integrated system embedded in its environment^6^. During walking, visual information is continuously sampled to guide foot placement, maintain balance, and anticipate upcoming terrain^7, 8, 9^. The eyes, head, and body therefore operate as a coordinated system that supports predictive control, allowing individuals to plan movements several steps ahead while adapting to immediate environmental demands^10, 11^. Examining gaze behaviour during locomotion thus provides insight into the strategies used to support safe and efficient movement.

Recent advances in wearable sensing have enabled researchers to investigate gaze, locomotion, and environmental context directly in natural environments. Mobile eye tracking, inertial sensors, and GPS-based localization now allow visual behaviour, movement dynamics, and environmental features to be measured simultaneously during everyday activities^12, 13^. These developments have facilitated a growing shift toward studying perception and action under ecologically valid conditions^14, 15, 16^.

While these advances have enabled the study of natural behaviour in real-world settings, much of our current understanding of vision and locomotion still originates from controlled laboratory research. These studies provided foundational insights into how gaze guides movement under precisely defined conditions. Early experiments showed that fixations are strategically directed toward future stepping locations, typically one to two steps ahead, revealing the feedforward nature of locomotor control^17, 18, 19^. Subsequent manipulations of visual preview confirmed that restricting the visible path beyond two steps increases stepping errors and reduces walking speed, underscoring the importance of predictive sampling for efficient gait planning^20^. Together, these findings demonstrate that gaze behaviour is not merely a passive record of visual salience but rather an anticipatory tool for coordinating upcoming movements. While visual sampling is shaped by bottom-up features such as surface irregularities or obstacles, it is also influenced by top-down factors related to navigation goals and intended walking trajectories. Yet laboratory walkways, though well controlled, simplify the spatial and sensory richness of real environments, leaving open how these mechanisms operate amid the irregular surfaces and dynamic obstacles of everyday life.

Studies using portable motion sensors have begun to characterize how locomotion adapts to environmental demands under natural conditions. Field studies using foot-mounted IMUs and metabolic monitoring have shown that uneven terrains increase energy expenditure and modulate gait parameters such as stride variability, foot clearance, and cadence^21, 22^. Experiments on multi-surface walkways likewise reveal that both young and older adults slow down and shorten their steps when negotiating irregular or slippery surfaces or when their vision of the lower visual field is obstructed, reflecting compensatory strategies for maintaining stability^23, 24^. These investigations have highlighted how locomotion adapts biomechanically to environmental demands. However, they primarily describe how people move, not how they allocate visual attention to guide those movements. Without concurrent measures of gaze, the perceptual and cognitive processes underlying these adaptations remain largely inferred.

Naturalistic eye-tracking studies have begun to address this gap by directly examining where people look while walking in complex environments. Early mobile recordings revealed that walkers direct a greater proportion of gaze toward the ground immediately ahead when transitioning from paved to uneven surfaces such as dirt trails^25^. Later comparisons between real-world and laboratory viewing showed that gaze patterns during actual walking are more centralized and stabilized, reflecting a coordinated use of head and eye movements to support balance and trajectory control^26, 27^. A landmark study by Matthis et al.^28^, further demonstrated that gaze distance systematically scales with terrain difficulty: on rough ground, gaze projections on the floor are closer to the body and more tightly coupled to upcoming footholds, whereas on flat surfaces they extend further ahead. These findings demonstrate that terrain systematically influences gaze behaviour and visual sampling during locomotion. However, much less is known about how concurrent adaptations in gait relate to these visual strategies during everyday urban walking. Moreover, most prior naturalistic studies examined a limited range of terrains, involved small samples, or focused on isolated gaze metrics, leaving the relationship between gaze and gait largely unexplored.

Despite these advances, three important gaps remain. First, most naturalistic studies have focused primarily on gaze behaviour or locomotion in isolation. Second, relatively little is known about how terrain-related adaptations in gaze and gait are coordinated during everyday urban walking. Third, few studies have spatially contextualized gaze and gait data within real-world urban environments, limiting our ability to identify where specific visuomotor adaptations emerge and how they relate to environmental structure.

Building on these theoretical and empirical advances, the present study investigated how terrain type shapes gaze behaviour and gait dynamics during naturalistic urban walking. Using wearable eye-tracking, foot-mounted inertial sensors, and GPS-based environmental contextualization, we examined how healthy young adults adapted their visual and locomotor behaviour while traversing a predefined 1.9 km urban walking route containing flat pavements, cobblestones, and dirt paths. Participants were instructed to follow the route while walking at a comfortable pace, allowing terrain-dependent adaptations in gaze and gait to be assessed under semi-naturalistic conditions. We hypothesized that increasingly irregular terrain would be associated with more cautious gait patterns and increased visual monitoring of the walking surface. We further examined whether terrain-related changes in gaze and gait covaried spatially across the route. By jointly characterizing gaze behaviour, gait dynamics, and their spatial coupling across common urban terrain types, this study extends previous work that primarily focused on gaze adaptations to terrain difficulty. In doing so, it provides one of the first spatially contextualized accounts of how terrain-dependent adaptations in gaze and gait emerge together during real-world urban walking.

## Results

We report behavioural results in three sections: (i) gaze behaviour across terrain types, (ii) gait dynamics across terrain types, and (iii) spatial relationships between gaze and gait features.

### Gaze dynamics across terrain types

We first examined how terrain influenced where participants looked while walking. Mobile eye-tracking provided continuous 2D gaze positions and calibrated estimates of gaze depth.

#### Horizontal and vertical gaze eccentricity distribution

**Fig. 1:**
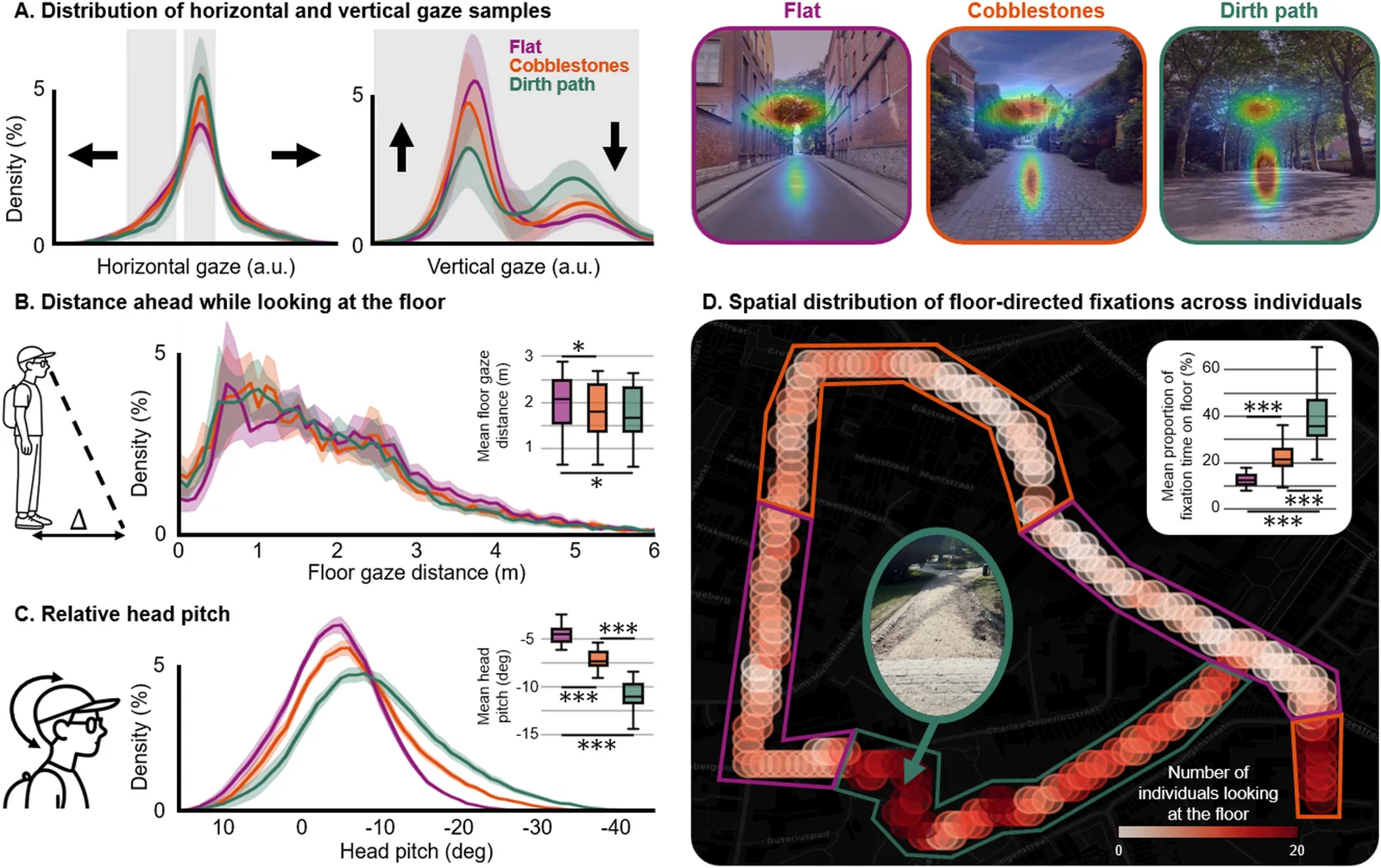
Gaze features across terrain types: flat (purple), cobblestones (orange), and dirt paths (green). (**A**) Grand-average (*N* = 20) kernel density plots of horizontal and vertical gaze-sample distributions per terrain, computed from normalized scene-camera coordinates (0–1 corresponding to the full width and height of the scene image). Density values indicate the relative distribution of gaze samples across spatial bins, normalized such that the total area under each curve sums to 100%. Grey bands mark x-axis bins with significant omnibus differences (*p* <.05) based on 1D cluster-based permutation tests (5000 permutations) with FDR correction applied (*q* < 0.05). Heatmaps show 2D gaze density per terrain (warmer colours indicate higher density). (**B**) Grand average density distributions of look-ahead distance during floor-directed fixations (m). (**C**) Grand average density distributions of head-pitch deviation (°) relative to a forward-looking baseline. (**D**) Spatial map of the grand-average floor-looking event density along the route; segments are coloured by terrain type. Example frames from locations with the highest floor-looking density are shown for illustration. Inset in panels B-D summarize participant-level mean values for each terrain condition; asterisks indicate significant pairwise differences.

The spatial distribution of gaze samples varied significantly with terrain type in both the horizontal and vertical planes. Density distributions along both axes were subjected to permutation-based cluster-level analyses (5000 permutations) with FDR correction (False Discovery Rate^29^) applied. In the horizontal plane, gaze distributions during dirt path walking exhibited significantly higher kurtosis compared to flat and cobblestone terrain, suggesting a narrower focus of visual attention. In the vertical plane, the overall gaze distribution across all terrain types was bimodal, with peaks corresponding to forward-facing (eye-level) and downward (floor-directed) viewing. On flat and cobblestone terrain, gaze was predominantly concentrated near the forward-facing mode. In contrast, during dirt path walking, gaze samples were more evenly distributed between the forward and downward peaks, indicating more frequent transitions between distant and near-ground visual monitoring (see Fig. 1A).

#### Floor-fixation distance

A significant main effect of terrain type was observed on the distance ahead at which participants fixated the floor, F(1.31, 25.01) = 4.70, *p* <.05, η² = 0.19. Post-hoc paired-sample t-tests showed that participants looked further ahead when walking on flat terrain compared to cobblestones, t(19) = 2.57, *p* <.05, d = 0.20, and dirt paths, t(19) = 2.72, *p* <.05, d = 0.28. No significant difference in floor-fixation distance was found between cobblestones and dirt paths, t(19) = 0.14, *p* =.89, d = 0.01 (see Fig. 1B).

#### Head pitch deviation from baseline

Terrain type had a significant effect on head pitch deviation relative to a forward-looking baseline, F(1.44, 27.48) = 67.61, *p* <.001, η² = 0.78. On flat terrain, participants maintained a head pitch angle comparable to the baseline, whereas their heads were significantly pitched downward when walking on cobblestones, t(19) = 5.38, *p* <.001, d = 0.92, and dirt paths, t(19) = 11.61, *p* <.001, d = 1.98. The downward pitch was significantly more pronounced on dirt paths than cobblestones, t(19) = 6.23, *p* <.001, d = 1.06 (see Fig. 1C).

#### Proportion of floor-directed fixation time

There was a robust main effect of terrain type on the proportion of time spent fixating the floor, *F*(1.32, 25.23) = 79.38, *p* <.001, η² = 0.80. Participants directed significantly more of their gaze toward the ground when walking on dirt paths (M = 39.4%, SD = 12.3) compared to cobblestones (M = 22.9%, SD = 6.4), *t*(19) = 7.82, *p* <.001, *d* = 2.00, and flat terrain (M = 13.0%, SD = 3.5), *t*(19) = 12.46, *p* <.001, *d* = 3.19. Floor-directed fixation time was also significantly higher during cobblestone walking than on flat terrain, *t*(19) = 31.26, *p* <.001, *d* = 8.57 (see Fig. 1D).

### Gait dynamics across terrain types

To quantify how locomotion adapted to terrain structure, we extracted pace, cadence, stride length, and stride duration from foot-mounted IMUs.

#### Walking pace

**Fig. 2:**
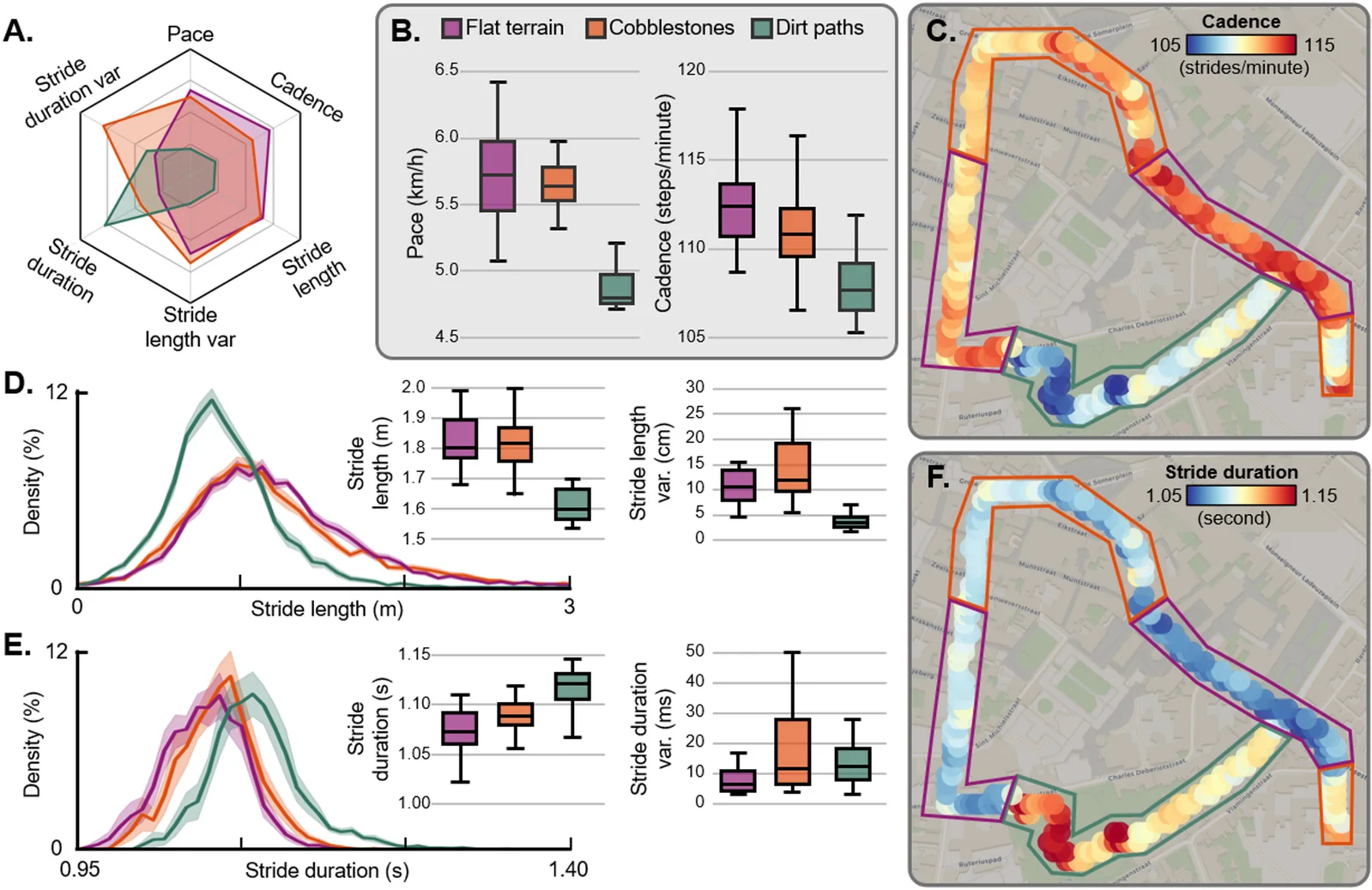
Gait features across terrain types: flat (purple), cobblestones (orange), and dirt paths (green). (**A**) Radar plot illustrating the group-level gait profile (*N* = 20) for each terrain, based on six gait metrics: walking pace (km/h), cadence (steps/min), stride length (m), stride duration (s), and the variability (standard deviation) of stride length and stride duration. Z-score normalization was applied across terrain values for each metric to enable direct visual comparison. (**B**) Boxplots of mean walking pace and cadence across terrain types. Asterisks indicate statistically significant pairwise differences (*p* <.05). (**C**) Spatial map of the walking route showing the grand average instantaneous cadence along the path; different segments are coloured according to terrain type. (**D**) Kernel density plots of stride length distributions per terrain; inset boxplots display group means and variability of stride length. Asterisks denote significant pairwise differences between terrain types (*p* <.05). (E) Kernel density plots of stride duration distributions per terrain; inset boxplots display group means and variability of stride duration. Asterisks indicate statistically significant pairwise comparisons (*p* <.05). (F) Spatial map of the walking route showing the grand average instantaneous stride duration along the path, with segment types coloured by terrain type.

A significant main effect of terrain type on average walking pace was observed, F(1.51, 28.82) = 66.76, *p* <.001, η² = 0.77. Post-hoc paired-sample t-tests revealed that participants walked significantly slower on dirt paths (M = 4.86 km/h, SD = 0.20) than on cobblestones (M = 5.70 km/h, SD = 0.43), t(19) = 9.33, *p* <.001, d = 2.12, and flat terrain (M = 5.81 km/h, SD = 0.48), t(19) = 10.56, *p* <.001, d = 2.40. Walking pace did not significantly differ between cobblestones and flat terrain, t(19) = 1.22, *p* =.22, d = 0.27 (see Fig. 2B).

#### Walking cadence

A significant main effect of terrain type on average cadence was found, F(2, 38) = 100.13, *p* <.001, η² = 0.84. Participants showed a higher cadence on flat terrain (M = 112.64 spm, SD = 2.58) compared to cobblestones (M = 111.18 spm, SD = 2.61), t(19) = 4.33, *p* <.001, d = 0.57, and dirt paths (M = 107.99 spm, SD = 2.42), t(19) = 13.83, *p* <.001, d = 1.82. Cadence was also significantly higher on cobblestones compared to dirt paths, t(19) = 9.49, *p* <.001, d = 1.25 (see Fig. 2B–C).

#### Stride length

There was a significant main effect of terrain type on stride length, F(1.55, 29.45) = 45.16, *p* <.001, η² = 0.70. Participants exhibited shorter stride lengths on dirt paths compared to cobblestones, t(19) = 8.41, *p* <.001, d = 2.11, and flat terrain, t(19) = 8.41, *p* <.001, d = 2.21. No significant difference was found between cobblestones and flat terrain, t(19) = 0.37, *p* =.70, d = 0.10 (see Fig. 2D).

#### Stride duration

A significant main effect of terrain type on stride duration was observed, F(2, 38) = 98.83, *p* <.001, η² = 0.83. Stride duration was shorter on flat terrain than on cobblestones, t(19) = 4.67, *p* <.001, d = 0.63, and dirt paths, t(19) = 13.82, *p* <.001, d = 1.87. Stride duration was also significantly shorter on cobblestones than dirt paths, t(19) = 9.14, *p* <.001, d = 1.24 (see Fig. 2E).

#### Stride variability

Variability was analysed separately for stride length and stride duration. A significant main effect of terrain type was found for stride duration variability, F(1.03, 19.58) = 7.43, *p* <.05, η² = 0.28, but not for stride length variability, F(1.01, 19.30) = 3.23, *p* =.09, η² = 0.14. However, post-hoc paired-sample t-tests did not reveal any significant pairwise differences in stride duration variability across terrains (see Fig. 2D–E).

### Spatial relationships between gait and gaze features

To examine whether different behavioural features tended to co-occur at the same locations along the route, we computed pairwise correlations between all gait, gaze, and head-orientation measures across spatial bins within each participant and then summarized these relationships at the group level using Fisher z-transformation (see Methods). Statistical significance was assessed via within-subject permutation testing (*N* = 5000, two-sided) and corrected for multiple comparisons using the Benjamini–Hochberg false discovery rate (FDR) procedure with q < 0.05.

#### Gait-gaze relationships

**Fig. 3:**
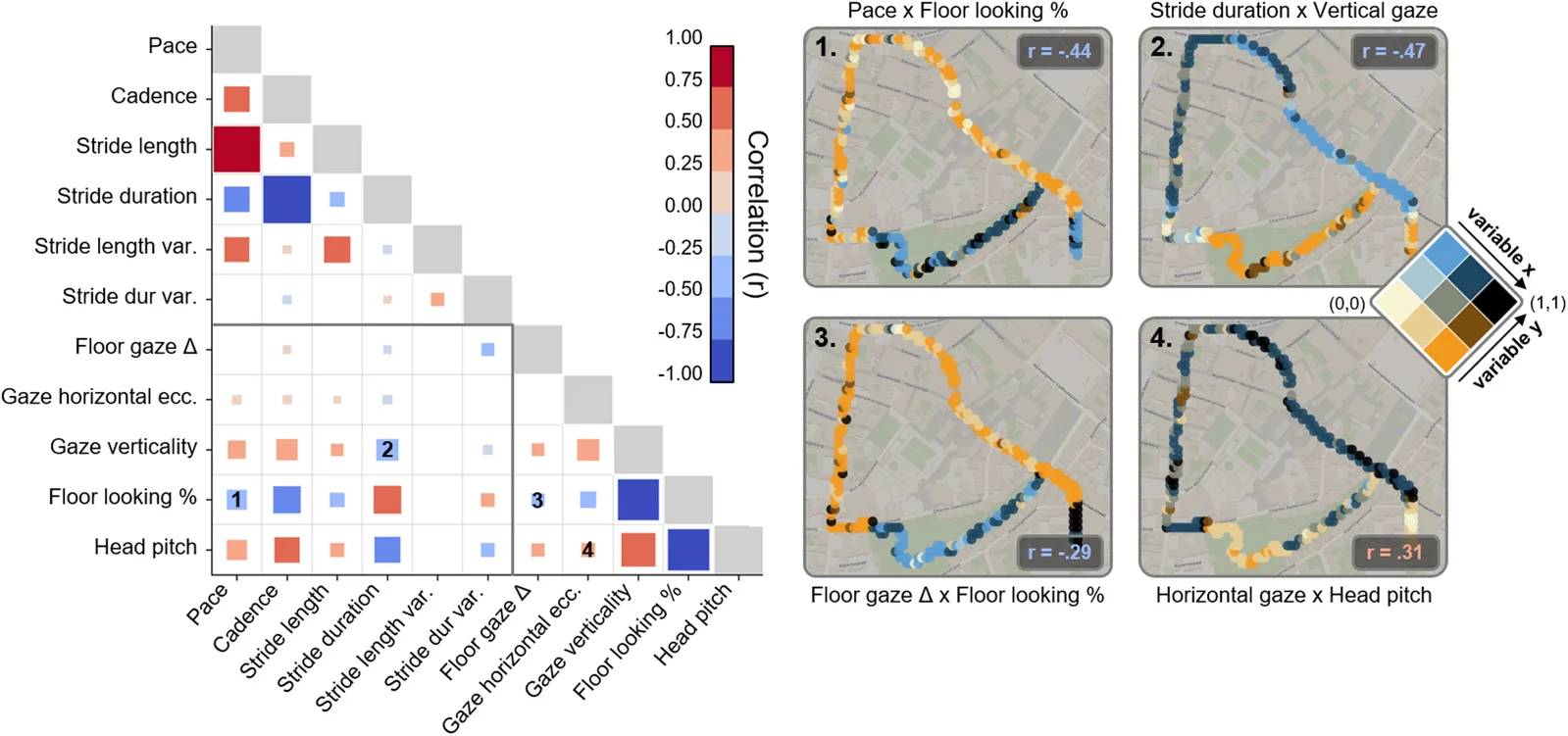
Group-level spatial-bin correlations between behavioural features. Correlations quantify whether behavioural features tended to co-occur at the same spatial locations along the route within participants. Left: Lower-triangle matrix of pairwise Spearman correlations computed within each participant across walking spatial bins, then aggregated across participants via Fisher z–mean and back-transformed to r. Colour encodes r using a discretized colormap (8 bins spanning − 1.00 to + 1.00); square size scales with |r| (larger squares indicate stronger associations). Cells with non-significant group effects (two-sided within-subject permutation test, 5000 permutations per pair; Benjamini–Hochberg FDR q < 0.05) are masked. The upper triangle is omitted for clarity; grey diagonal squares denote unity (*r* = 1). Gait features: walking pace, cadence, stride length, stride duration, stride-length variability, and stride-duration variability. Gaze/head features: absolute horizontal eccentricity and gaze verticality (higher values = upper visual field), floor-level look-ahead distance (depth averaged during floor-looking only), proportion of floor-looking samples, and head-pitch deviation from a look-ahead baseline (negative = downward tilt). A grey rectangle highlights the cluster of gait × gaze feature correlations. Right: Four subject-balanced bivariate maps illustrating the spatial co-variation of selected gait and gaze feature pairs, corresponding to the numbered entries in the group-level correlation matrix (left). Pairs: (1) walking pace × floor-looking %, (2) stride duration × gaze verticality, (3) floor gaze distance × floor-looking %, (4) horizontal gaze eccentricity × head pitch. Spatial relationships are visualized using a 3 × 3 bivariate colormap representing joint levels.

Several significant FDR-corrected gait × gaze associations emerged (Fig. 3, left). Walking pace was positively correlated with gaze verticality (higher values indicating more upward gaze positions within the scene camera) (*r* =.39, *p* <.001), head pitch (*r* =.43, *p* <.001), and horizontal gaze eccentricity (*r* =.22, *p* <.001), and negatively correlated with the proportion of floor-looking (*r* = −.44, *p* <.001). These results indicate that faster walking was associated with more upward and outward gaze and reduced monitoring of the ground.

Cadence showed a similar pattern, with even stronger correlations to head pitch and gaze verticality, and an additional positive correlation with gaze depth (*r* =.20, *p* <.001). Stride length also followed this trend, being positively associated with all gaze features aligned with faster walking. Higher step frequency and longer strides similarly co-occurred with looking further ahead.

In contrast, stride duration showed the opposite pattern: it was negatively correlated with head pitch (*r* = −.55, *p* <.001), gaze verticality (*r* = −.47, *p* <.001), horizontal eccentricity (*r* = −.22, *p* <.001), and gaze depth (*r* = −.20, *p* <.001), and positively associated with floor-looking proportion (*r* =.59, *p* <.001). Longer stride durations were linked to more downward and centralized gaze, reflecting more cautious locomotion.

While stride length variability showed no significant relationships with gaze features, stride duration variability was positively associated with floor-looking (*r* =.30, *p* <.001), and negatively with gaze depth (*r* = −.28, *p* <.001), gaze verticality (*r* = −.23, *p* <.001), and head pitch (*r* = −.31, *p* <.001). More variable stride duration was observed in locations where gaze was directed downward and close.

#### Gait feature intercorrelations

As expected, gait features were strongly interrelated. Walking pace was positively correlated with cadence (*r* =.54, *p* <.001) and stride length (*r* =.95, *p* <.001), and negatively with stride duration (*r* = −.54, *p* <.001). Cadence was inversely correlated with stride duration (*r* = −.98, *p* <.001), and positively with stride length (*r* =.20, *p* <.001). Stride length variability was also positively associated with pace (*r* =.52, *p* <.001), cadence (*r* =.20, *p* <.001), and stride length (*r* =.56, *p* <.001). These correlations reflect the expected biomechanical coupling between gait metrics.

#### Gaze feature intercorrelations

Several gaze features showed strong intercorrelations, as expected given their shared role in characterizing viewing direction and floor-looking behaviour. Notably, both gaze verticality and head pitch were positively associated with horizontal gaze eccentricity (*r* =.46 and *r* =.31, respectively; both *p* <.001), suggesting that a broader horizontal distribution of gaze occurred when individuals oriented their head and gaze upward. Gaze depth was also positively correlated with both gaze verticality and head pitch (*r* =.29 for both, *p* <.001), indicating that looking further ahead in space tended to co-occur with upward viewing angles. In contrast, horizontal eccentricity was negatively associated with the proportion of floor-looking (*r* = −.35, *p* <.001), suggesting that gaze became more centrally focused when individuals looked downward toward the floor.

## Discussion

This study examined how variations in urban terrain shape visuomotor behaviour during real-world walking. Across multiple gaze and gait measures, we observed a consistent gradient of adaptation: compared with flat pavements, increasingly irregular terrains elicited more ground-directed visual monitoring and a slower, more cautious gait pattern. These findings indicate that gaze and locomotor behaviour are flexibly coordinated in response to environmental demands.

On flat terrain, participants adopted a forward-facing visual strategy characterized by narrowly centred gaze distributions, minimal downward head pitch, and a low proportion of floor-directed fixations. In contrast, irregular terrains, particularly dirt paths, elicited pronounced shifts toward downward and proximal gaze, along with greater head-pitch deviation, indicating increased visual monitoring of the ground. These findings are consistent with previous observations that walkers direct their gaze closer to the body when negotiating uneven terrain^25, 28^. Cobblestones produced intermediate effects, suggesting that even structured yet uneven surfaces evoke more cautious visual strategies. Together, the results extend laboratory findings on obstacle negotiation^23, 24^ to real-world settings, demonstrating that gaze behaviour and visual sampling are strongly influenced by terrain complexity. Participant-level measures (see Supplementary Figures S1–S3) further indicate that these terrain-related adaptations were broadly consistent across individuals despite natural inter-individual variability.

However, terrain characteristics are unlikely to be the only factor contributing to these gaze adaptations. An additional possibility is that the observed patterns reflect broader differences in the visual environment. The dirt-path segments were located within park-like areas that contained fewer buildings, signs, and pedestrian interactions than the urban street segments. Consequently, participants may have devoted less visual sampling to the surrounding environment and more to monitoring the walking surface itself. Although the present design does not allow these influences to be disentangled, this observation highlights the importance of considering both terrain properties and the wider visual environment when interpreting gaze behaviour during natural locomotion.

Gait dynamics showed a parallel gradient of adaptation. Participants walked more slowly on irregular surfaces, with shorter strides, longer stride durations, and reduced cadence, consistent with a conservative gait strategy that prioritizes stability over efficiency^30^. Flat terrain supported faster, more rhythmic walking, whereas cobblestones again produced intermediate adjustments. Together, these findings suggest that terrain complexity shapes not only visual sampling behaviour but also the timing and organization of locomotion itself.

Most importantly, terrain-related changes in gaze and gait did not occur independently but were tightly coupled. On dirt paths, slower pace and greater stride-duration variability were accompanied by more downward-directed gaze and shorter look-ahead distances, suggesting increased reliance on visual information to guide movement. Conversely, faster and more efficient walking on flat terrain coincided with more elevated gaze and reduced ground monitoring. Spatial correlation analyses reinforced this reciprocal pattern, revealing that faster walking and longer strides were consistently associated with more upward and outward gaze, whereas slower or more variable gait was associated with increased near-field monitoring. Together, these findings suggest that visual sampling and locomotor control are coordinated components of an adaptive visuomotor system that continuously responds to environmental demands.

Beyond the behavioural findings, this study demonstrates the value of combining wearable eye-tracking, inertial sensing, and GPS-based environmental contextualization to investigate visuomotor behaviour in real-world settings. By linking continuous gaze and gait recordings to specific terrain categories, we were able to relate behavioural adaptations directly to the physical structure of the environment. This spatially contextualized approach extends previous studies that examined gaze or locomotion in isolation by enabling the joint characterization of visual and locomotor adaptations across everyday urban terrains.

More broadly, the framework provides a foundation for moving beyond discrete environmental categories toward continuous descriptions of environmental structure. Future work could integrate additional spatial features, such as slope, surface roughness, spatial configuration^31^, or computer-vision-derived scene descriptors^32, 33^, to investigate how fine-grained variations in the environment shape visuomotor behaviour.

Floor-looking distance was estimated using a participant-specific calibration-based vergence approach^34^. Although this method provides a practical estimate of gaze depth under natural viewing conditions, vergence-based measures remain sensitive to noise, particularly during locomotion and at longer viewing distances^35^. Real-world mobile eye-tracking also introduces practical challenges that are rarely encountered in laboratory research, including sunlight interference with infrared pupil tracking, heat generated by recording units, and changes in ambient lighting that may affect computer-vision processing (Nolte, et al., 2025). These issues were mitigated by scheduling recording sessions under suitable weather conditions, providing participants with lightweight caps to shade the eye-tracking sensors, and using participant-specific calibration procedures. Nevertheless, terrain-related differences in look-ahead distance should be interpreted with appropriate caution and may represent conservative estimates of the underlying perceptual adaptations. Future work could leverage emerging deep-learning and photogrammetric approaches to improve the robustness and range of depth estimation in naturalistic environments^36^.

The present analyses focused on gait measures that are commonly used to characterize locomotor adaptations to different terrain types, including walking pace, cadence, stride length, stride duration, and their variability. Because gait was recorded using a single foot-mounted IMU, we could not assess interlimb coordination metrics such as relative phase. Future studies using bilateral foot instrumentation could determine whether terrain complexity also influences coordination between the limbs, providing a more comprehensive characterization of locomotor control.

Although the present protocol took place in real-world urban environments, certain aspects of the experimental setup inevitably shaped participants’ behaviour. The presence of an experimenter walking a few metres behind may have introduced a mild social or monitoring effect, potentially influencing how naturally participants behaved. Likewise, wearing eye-tracking glasses connected to a backpack could have increased self-awareness and subtly constrained head movements. In addition, the task itself was partially scripted: participants followed a predefined route rather than navigating spontaneously or engaging in their own everyday activities. This design choice was intentional, as it reduced variability in route choice and navigational demands, allowing us to isolate the influence of terrain characteristics on gaze and gait behaviour. However, it also constrained an important aspect of natural urban locomotion, namely the autonomy to select routes, make wayfinding decisions, and visually explore alternative paths. Consequently, the present findings should be interpreted as reflecting visuomotor adaptations during route execution rather than route planning or navigational decision-making. Future studies could extend the present multimodal framework to more open-ended navigation paradigms, allowing researchers to examine how environmental structure influences not only locomotion and visual attention during movement, but also the route-selection processes that shape everyday navigation.

While our results underscore the tight coordination between perception and action, one limitation is that terrain-related gaze differences may be partly driven by concurrent changes in walking speed. Slower movement allows more time for ground monitoring, whereas faster locomotion naturally promotes forward-looking gaze. Although our spatial correlation analyses revealed a consistent coupling between gaze and gait, future work should formally model speed as a potential mediator to determine the extent to which gaze adaptations reflect direct responses to terrain or indirect consequences of gait modulation.

Despite these considerations, the observed coupling between gaze behaviour and locomotor behaviour is broadly consistent with theoretical models that treat locomotion as a perception-action cycle^7^, in which visual information is actively sampled to guide movement. Rather than operating independently, perceptual and motor processes appear to adapt together in response to environmental demands, extending classical theories of visual guidance of locomotion^9, 17, 18^ by demonstrating that these predictive gaze strategies operate continuously in natural urban environments, even in the absence of explicit obstacles or targets. However, it is important to note that gaze direction should not be equated with attentional allocation. Although eye-tracking provides a valuable window into the visual information sampled during locomotion, attention can also be allocated covertly to locations outside the current point of gaze. Accordingly, interpretations regarding visuospatial attention should be understood as inferences based on observed gaze behaviour.

The present findings also contribute to the growing effort to study behaviour in the environments where it naturally unfolds^37, 38, 39^. While real-world studies inevitably sacrifice some experimental control, they provide insight into how perception and action operate under the complex and dynamic conditions of everyday life, where behaviour must continuously adapt to changing contextual demands^40, 41^. In this regard, laboratory and field studies should be viewed as complementary approaches: controlled experiments isolate specific mechanisms, whereas naturalistic studies reveal how those mechanisms interact with contextual factors^16, 42^. The present study occupies an intermediate position within this continuum, combining the ecological validity of real-world walking with a partially standardized route that enabled the systematic examination of terrain-related adaptations in gaze and gait.

Because data were collected in a naturalistic urban environment, pedestrian density varied across recording sessions and was not experimentally controlled. Social context may therefore have contributed to some of the observed terrain-related effects. Previous work suggests that the presence of other pedestrians can influence both visual exploration and locomotor behaviour^43, 44^. Future studies explicitly manipulating or quantifying social density alongside terrain characteristics would help disentangle the relative contributions of physical and social environmental factors to visuomotor behaviour. Other contextual factors, such as time of day, lighting conditions, and auditory soundscape, may similarly influence visuomotor behaviour and warrant investigation in future studies.

Although the participants in this study were healthy young adults, the terrain-dependent adaptations observed here highlight the perceptual and motor processes that support safe locomotion in everyday environments^45^. In populations with visual, vestibular, or attentional impairments, these adaptive mechanisms may become less effective, contributing to instability and increased fall risk^46^. Previous work has shown that older adults adopt different visual strategies when negotiating stairs and obstacles, often increasing ground monitoring to compensate for reduced predictive control^47, 48, 49^. Extending the present approach to populations with neurological or sensory impairments, such as Parkinson’s disease, stroke, or glaucoma, could provide insight into how visuomotor adaptations are altered under natural walking conditions^50, 51, 52^.

Mapping the spatial co-variation of gaze and gait revealed not only terrain-level differences, but also localized micro-adaptations associated with specific environmental irregularities. These spatially anchored patterns capture how the built environment dynamically shapes visuomotor behaviour, highlighting locations where perceptual or locomotor demands increase.

Such findings may be relevant for inclusive urban design. Locations that consistently elicit downward gaze, reduced walking speed, or increased stride variability may indicate subtle environmental challenges that are manageable for healthy adults but potentially problematic for individuals with sensory, cognitive, or mobility impairments. Spatially contextualized behavioural measures could therefore complement traditional infrastructure assessments by revealing how people actually experience and navigate urban environments.

More broadly, the present results suggest that gaze behaviour and gait dynamics provide sensitive indicators of terrain-related environmental demand. Integrating these measures into urban analyses may help identify locations where design features increase perceptual or locomotor burden, thereby informing targeted interventions aimed at improving accessibility, safety, and walkability.

Urban walking may feel effortless, yet our findings show that it is supported by systematic, terrain-dependent reconfiguration of both where people look and how they move. Compared with flat pavements, dirt paths prompted more frequent ground-directed fixations at shorter look-ahead distances, larger downward head-pitch deviations, and a slower, shorter-stride, longer-duration gait pattern. Cobblestones produced intermediate adaptations along the same dimensions. Crucially, spatial analyses of gaze-gait covariation revealed that elevated, outward gaze tended to coincide with faster pace and longer strides, whereas downward, proximal gaze was linked with slower and more variable stepping.

By linking eye, head, foot, and environmental data collected in real-world settings, this study demonstrates that everyday locomotion relies on continuous visuomotor coordination that adapts to environmental structure. More broadly, the findings show how combining wearable sensing with spatial contextualization can reveal the dynamic interplay between visual sampling and locomotor control during natural behaviour. Together, these results provide a spatially grounded account of terrain-dependent visuomotor adaptation in urban environments. This framework may also provide a foundation for future investigations of locomotion in diverse populations and for the development of more human-centred approaches to urban design.

## Methods

### Participants

Twenty self-reported righthanded adults took part in the study (11 women; mean age = 26.5 years, SD = 5.18, range = 20–37). Two datasets were excluded due to a synchronization issue during one of the sessions; replacement participants were recruited to maintain the planned sample size. All participants reported normal or corrected-to-normal vision, no motor impairments, and no medical or neurological conditions. Participants were recruited via the Faculty of Psychology and Educational Sciences (KU Leuven) research participation platform. Before testing, they were informed about the procedures and data handling; all provided written informed consent and were reminded of their right to withdraw at any time without justification. The study adhered to the Declaration of Helsinki and was approved by the KU Leuven Social and Societal Ethics Committee (SMEC; approval G20259662).

### Sample size justification

Given the novelty of our paradigm and the absence of directly comparable prior work, we conducted a post hoc sensitivity analysis based on our primary outcome measures: the proportion of time spent fixating the floor and the look-ahead distance during floor-directed fixations. The effect of terrain on fixation proportion was very large (η²ₚ = 0.80, f = 2.00), and the effect on look-ahead distance was also large (η²ₚ = 0.19, f = 0.48). For a within-subjects design with three levels (α = 0.05, *N*= 20), sensitivity analysis indicated that effects of f ≥ 0.25 could be reliably detected with 95% power. Both observed effects therefore exceeded the detectable threshold by a substantial margin. This aligns with the magnitude of terrain-related differences reported by Matthis et al.^28^, who compared look-ahead distance across three surface types. Accordingly, our study was well-powered *a posteriori*, and the final sample of 20 participants provided excellent sensitivity (> 0.99) to detect effects of comparable or larger size, supporting the robustness of the observed terrain differences.

### Procedure

**Fig. 4:**
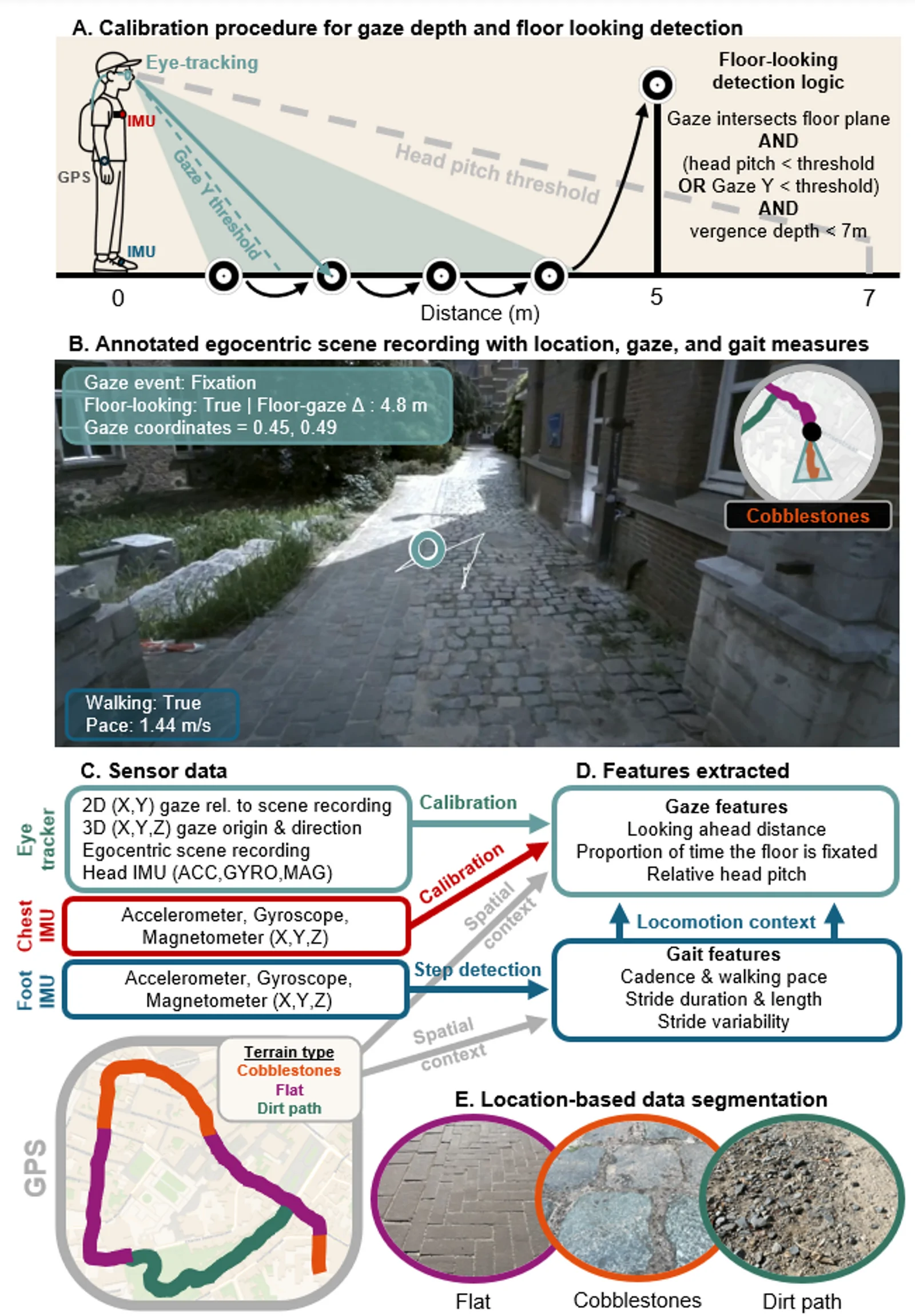
(A) Schematic representation of the sensors placement and procedure used to calibrate gaze depth estimates based on vergence measures and for the definition of adaptive thresholds for the detection of floor-looking. (**B**) Annotated frame of the egocentric scene recording of one participant illustrating simultaneous data streams recorded (gaze, gait, location) and their associated features extracted. (**C**) List of raw data features recorded across sensors (eye-tracking, IMUs, GPS). (**D**) Computed measures derived from sensor data. The arrows indicate the different types of sensor data that contribute to the computation of the extracted features. (**E**) The continuous data was segmented based on terrain type which was retrieved from location data.

Participants were greeted by the experimenter and received verbal and written information about the study. After providing informed consent, their height was measured to support subsequent gaze calibration. The IMU-based motion sensors and eye-tracking glasses were then fitted. Sessions were scheduled under suitable weather conditions and participants wore lightweight caps to shield the eye-tracking sensors from sunlight, in line with established recommendations for outdoor mobile eye-tracking^53^. Participants were shown a map of the 1.9 km predefined walking route. The experimenter highlighted key segments and intersections, using prominent landmarks to aid orientation, thereby providing participants with a clear top-down navigational goal that was consistent across subjects. All participants were either KU Leuven students or staff and reported being familiar with the city. The route was located in a frequented urban area of Leuven, and participants walked exclusively on pedestrian-only streets or sidewalks throughout. Data collection took place at various times of day, resulting in natural variation in pedestrian density across sessions, consistent with the naturalistic design of the study. While participants were not permitted to use a phone or map during the walk, they were informed that the experimenter walking a few meters behind would discretely provide guidance if they became disoriented. Such intervention was rarely required, occurring once for five participants and twice for two participants. Before the main task, participants completed a brief floor-looking distance calibration (see Fig. 4A). This involved fixating four visual targets placed on the ground at increasing distances (1–4 m), as well as one target at eye level located 5 m ahead, all while maintaining a natural forward head posture. This procedure was used to calibrate individual gaze distance estimates based on vergence, and to define participant-specific thresholds for distinguishing floor-directed gaze and head pitch variations. The participant and experimenter then proceeded to the starting location. Once the smartwatch recording was initiated, participants began the walk independently, following the predefined route. Upon completion, the recording was stopped and saved. The participant and experimenter returned to the lab, the sensors were removed, and participants were debriefed.

### Materials

#### Eye-tracking

Participants’ gaze was recorded using Tobii Pro Glasses 3 eye-tracking glasses (Tobii, Stockholm, Sweden). The glasses were individually fitted for comfort, with adjustable nose pads and a soft securing lanyard. The system employed four infrared sensors (two per eye) that used corneal reflection, dark pupil detection, and stereo geometry to track eye movements at a sampling rate of 100 Hz. Integrated gyroscope and accelerometer sensors recorded head motion at the same rate (100 Hz). Gaze and head motion data were streamed in real time to the Lab Streaming Layer (LSL, Kothe et al.,^54^) framework for multimodal synchronization. To enable continuous streaming, the eye-tracking system was connected via Ethernet cable to a dedicated laptop running the LSL host. A front-facing scene camera embedded in the glasses recorded first-person perspective video at 25 frames per second (1920 × 1080 resolution; 95° horizontal/63° vertical field of view). These video recordings were stored internally on the glasses, as the video stream was not compatible with the LSL synchronization protocol. The Tobii system provided battery life of approximately one hour, which was sufficient to complete both the calibration phase and the full walking session.

#### Motion sensing

Torso and left foot motion were captured using two wearable EmotiBit modules (OpenBCI, New York, USA), each equipped with a BMI160 16-bit Inertial Measurement Unit (Bosch) and a BMM150 digital geomagnetic sensor (Bosch). One module was secured to the torso at the infrasternal angle (inferior end of the sternum) using an adjustable elastic belt. The second module was affixed to the left foot, positioned over the dorsal aspect of the talar dome. Each IMU recorded tri-axial accelerometer (linear acceleration), gyroscope (angular velocity), and magnetometry (magnetic field strength) along the X, Y, and Z axes at a sampling rate of 25 Hz. Motion data were wirelessly streamed to the LSL synchronization framework via a secure local Wi-Fi network hosted by the LSL computer.

#### Global positioning system

Participant geolocation was tracked using a Garmin VIVOACTIVE 4 S GPS smartwatch (Garmin Ltd., Switzerland). The device continuously recorded timestamped latitude, longitude, and elevation values during the outdoor walking session, stored in GPX format. These data provided a global reference for spatial localization and were later synchronized with the gaze and motion sensor recordings (see Sect. 2.4.2 for processing and alignment procedures).

### Data processing and analysis

#### Multimodal data alignment

The data streams from the eye-tracking glasses (gaze and head motion) and the torso-mounted motion sensor were synchronized via the LSL framework and saved into an Extensible Data Format (.xdf) file. Egocentric scene video recordings, along with synchronized gaze and head motion data, were stored locally on the eye-tracking glasses, as the video stream was not compatible with LSL. Because all devices were manually started by the experimenter, and because the LSL recording process involved variable startup delays across streams, the resulting.xdf file was both temporally offset and shorter in duration than the internally stored gaze/video recordings. To correct this, we first zero-padded the onset of the LSL-based gaze signal to match the duration of the internally stored gaze signal from the eye-tracking system. We then calculated the cross-correlation between the two gaze signals (based on normalized horizontal gaze positions) to identify the temporal lag associated with the highest correlation, which indicated optimal alignment. This lag was used to shift the LSL-based gaze data accordingly. If, after applying the lag, the realigned LSL gaze data started after time zero, additional zero-padding was inserted at the beginning of the LSL data. Similarly, if the aligned signal was still shorter than the internal gaze recording, zero-padding was added at the end to ensure consistent signal length. This procedure provided accurate alignment between LSL-based and eye-tracker-internal data streams. The added zero-padding only affected short periods before or after the actual walking task and did not influence downstream analyses of gaze or body motion features.

#### Geospatial processing and environmental annotation of gaze and gait data

GPS data were parsed from the recorded GPX files and pre-processed to generate a uniformly sampled trajectory at 1 Hz. Latitude, longitude, and elevation values were linearly interpolated to fill small temporal gaps, and cumulative walking distance was computed using the haversine formula between consecutive coordinates. Walking speed (km/h) was estimated from the distance profile using a 10-second rolling window to smooth instantaneous fluctuations.

To annotate the walking environment, each GPS coordinate was spatially joined with labelled polygonal regions defined in a custom GeoJSON file (available in the online repository: https://github.com/sladouce/LocoGaze_UrbanTerrainTypes*)*, corresponding to terrain categories such as flat pavement, cobblestones, and dirt paths. The positional accuracy of the smartwatch GPS is typically within a few metres under open-sky conditions, which can cause transient coordinate jitter near terrain boundaries. To reduce spurious label changes due to this jitter, a centred 5-second majority-vote filter was applied to the terrain time series, such that the final label for each 1 Hz sample corresponded to the most frequent terrain category within the surrounding ± 2 s.

A lag correction derived from synchronization metadata was then applied to align GPS timestamps with the other sensor streams (e.g., LSL-recorded IMU and gaze data). Finally, GPS data were temporally aligned to the moment of video onset, based on the video frame where the smartwatch recording began. The resulting annotated trajectory including position, instantaneous speed, and terrain labels was exported as a labelled CSV file.

#### Gaze behaviour during walking

To describe where participants directed their gaze within the egocentric scene while walking, we analysed the horizontal and vertical 2D gaze positions mapped onto the video frame (1920 × 1080 pixels). We selected all timepoints labelled as walking and grouped the samples by terrain (flat pavements, cobblestones, dirt paths). For each participant and terrain, gaze eccentricity values were binned into 20-pixel increments along each axis and converted into percentage-based histograms. These per-bin percentages (summing to 100%) captured the relative spatial distribution of gaze and were stored for group-level visualization and further analysis.

Gaze depth was estimated using a vergence-based approach. For each timestamp, binocular 3D gaze direction vectors were used to compute the angular difference between the eyes^34^, and the vergence distance was estimated using a standard inter-pupillary distance (IPD) formula^35^. Low-confidence samples (dot product ≤ 0.1) were excluded. The resulting gaze depth signal was then linearly calibrated using floor targets presented at 1 and 4 m distances from participant’s feet during controlled calibration trials of 10 s each. Median vergence values in these windows were mapped to the true distances, ensuring a participant-specific depth calibration. The final signal was smoothed with a Savitzky–Golay filter and yielded a continuous calibrated estimate of gaze depth (in mm).

To quantify how far ahead participants typically looked when fixating the ground, we selected timepoints classified as floor-directed gaze (see below). For each participant and terrain, depth values were grouped into 100 mm bins spanning 0–10,000 mm; values above 10,000 mm were capped. To compute a summary value, we restricted the analysis range to 0–7000 mm and calculated a weighted mean gaze depth, using histogram bin left-edges as the values and bin percentages as weights. This produced a single number per participant and terrain representing typical floor-directed fixation distance.

To classify whether participants were looking at the ground, we combined multiple gaze and posture indicators. First, a geometric ray-plane intersection was computed for each frame by intersecting the averaged binocular gaze ray with a horizontal plane placed at the estimated eye height (scaled from participant stature). Floor-directed gaze was defined based on whether this ray intersected the ground in front of the participant and was pointed sufficiently downward. Additional cues included: (i) downward head pitch relative to the torso (from head–torso IMU angular difference), (ii) vertical gaze pitch (derived from the 3D gaze vector), (iii) 2D vertical gaze coordinates located in the lower portion of the scene video frame, and (iv) calibrated gaze depth shorter than 7 m.

A timepoint was labelled as floor-looking if any of the following were true: (a) the smoothed head pitch was below a participant-specific dynamic threshold (derived from central and far-directed calibration periods); (b) a valid floor intersection occurred and the head pitch was low; or (c) the 2D gaze Y-coordinate exceeded 60% of the frame height. All criteria required the gaze depth to be < 7 m to exclude far fixations. The resulting binary floor-looking label was saved alongside the depth and head pitch measures for each frame.

To quantify the relative amount of time participants spent looking at the floor, we computed, for each participant and terrain, the proportion of walking frames classified as floor-looking. This was calculated as the number of floor-labelled samples divided by the total number of valid walking samples in that terrain, expressed as a percentage.

#### Head orientation during walking

Head pitch was computed as the angular difference between the head and torso IMUs in the sagittal plane. For each IMU, pitch was derived using the arctangent of the vertical and longitudinal acceleration axes, and the head-relative-to-body value was calculated by subtracting torso pitch from head pitch. This time series was baseline-corrected per participant using the average pitch measured during a central calibration window (5 m target at eye level). To assess terrain-related deviations, we summarized the head pitch signal for each terrain using histograms (− 45° to + 25° in 1° bins) and calculated weighted means (bin edges × bin percentages), yielding one average pitch value per participant and terrain.

#### Gait dynamics during walking

Gait features were extracted from tri-axial accelerometer and gyroscope signals recorded at the feet using a custom signal processing pipeline. Walking segments were first identified using the root mean square (RMS) of the acceleration magnitude over 1-second windows, applying a participant-specific threshold. Prior to gait event detection, the foot-IMU accelerometer magnitude signal was smoothed using a gaussian filter (sigma = 1 sample) to attenuate high-frequency noise and improve the reliability of peak detection. Stride events were then detected via peak finding on vertical acceleration, and stride durations were calculated from inter-peak intervals. Stride and step lengths were estimated using an inverted pendulum model, where angular displacement (from gyroscope integration) was converted to spatial displacement based on leg length. Cadence was estimated from the step intervals, and walking pace was calculated as the product of step length and cadence. To correct for potential drift in the IMU-derived speed estimates, the average walking speed from the IMU was scaled to match the GPS-derived average speed over the same period.

For analysis, we selected only the timepoints corresponding to walking and grouped all gait samples by terrain type (flat pavements, cobblestones, dirt paths). For each participant and terrain, we computed the mean walking pace (in km/h), cadence (in steps per minute), stride length (in meters), and stride duration (in seconds). To quantify gait stability, we additionally computed the sample variance of stride length and stride duration for each participant and terrain. These per-participant summaries were stored in terrain-labelled tables and used in subsequent group-level comparisons and visualization.

#### Statistical analysis of terrain-related differences in gaze and gait

To compare gaze and gait features across terrain types, we used one-way repeated-measures ANOVAs with terrain (flat pavements, cobblestones, dirt paths) as the within-subject factor. Where Mauchly’s test indicated a violation of sphericity, degrees of freedom were corrected using the Greenhouse–Geisser epsilon. Effect sizes were reported as partial eta-squared (η²). Significant main effects were followed up with pairwise post-hoc comparisons using paired-sample t-tests, with Cohen’s d reported as the effect size measure. All p-values from post-hoc tests were corrected for multiple comparisons using the Bonferroni method. The alpha threshold for statistical significance was set at *p* <.05 for all tests.

#### Correlations between gaze and gait features across spatial bins

As a hypothesis-guided exploratory analysis, we investigated how gaze and gait covary while individuals moved through the environment by computing pairwise correlations on spatially aggregated features. Because the objective of this analysis was to characterize spatial rather than temporal organization, measures were first aggregated within spatial bins before correlations were computed. For each participant, we first restricted the data to walking frames and then grouped samples into spatial bins by rounding GPS latitude and longitude to four decimals, yielding bins on the order of tens of metres. To ensure stable estimates, we retained only bins with ≥ 25 samples (i.e., a minimum of one second of data). All analyses in this section were performed at the bin level (one row per spatial bin per participant).

For each bin, we computed the mean of gait and gaze variables. Gait features included cadence (steps/min), pace (km/h), stride length (m), stride duration (s), and the within-bin variances of the latter two. Gaze features included absolute gaze verticality (inverted so that larger values indicate upward gaze) and horizontal eccentricity, defined as the absolute deviation from screen center, |gaze_x_ − 960| (in pixels). This eccentricity measure was applied only to the horizontal axis to capture the extent, but not the direction, of lateral gaze shifts which are typically symmetrically distributed around centre. In contrast, gaze verticality was retained in its signed form to preserve directionality, given its bimodal distribution and functional significance. We also computed the fraction of floor-looking within the bin (binary detector averaged over samples) and gaze depth (meters), obtained by averaging the calibrated vergence-based depth during floor-looking samples only. The deviation in head pitch from a look-ahead baseline (in degrees, with negative values indicating downward tilt) was also included.

Within each participant’s spatially binned dataset, we computed Spearman rank correlations between all available feature pairs across bins (using pairwise deletion for missing values). To summarize effects across participants while preserving effect-size scale, we applied a Fisher z-transform to each subject’s correlation, averaged z-values across participants with equal weighting, and back-transformed to *r* to yield the group correlation matrix.

Because the Fisher z-mean does not provide inferential statistics, we estimated group-level p-values using a within-subject permutation test for each feature pair. For each subject, one variable was randomly shuffled across bins (preserving its marginal distribution and the other variable), a new Spearman correlation was computed, transformed to z, and averaged across subjects. Repeating this 5000 times yielded a null distribution; the two-sided p-value equalled the proportion of permuted |z| values greater than or equal to the observed |z|.

To control for multiple comparisons, group-level permutation p-values were corrected using the Benjamini–Hochberg false discovery rate (FDR) procedure^29^ with a q < 0.05 threshold. FDR correction was applied jointly across all feature pairs within each matrix (and, for terrain-specific analyses, across all terrain matrices combined). Cells exceeding the FDR threshold were masked in all reported matrices and figures.

## Supplementary Information

Below is the link to the electronic supplementary material.

Supplementary Material 1

## Data Availability

The anonymized data are hosted in an open-access OSF repository (https://osf.io/hqasr). The code for data processing and analysis is freely available on GitHub (https://github.com/sladouce/LocoGaze_UrbanTerrainTypes).

## Code availability

The code for data processing and analysis is freely available on GitHub (https://github.com/sladouce/LocoGaze_UrbanTerrainTypes).
