# Supplementary material for: Gaze and gait dynamics are shaped by terrain type during urban walking
Source: Sci Rep. 2026 Jun 17;16:27733. doi: 10.1038/s41598-026-57119-8 (PMC13542172; doi:10.1038/s41598-026-57119-8)
Supplement: Supplementary file 1 [file 41598_2026_57119_MOESM1_ESM.pdf]

Supplementary Materials

The following supplementary figures provide participant-level visualizations of gaze distributions and paired gaze/gait measures across terrain conditions. These figures complement the group-level analyses presented in the main manuscript by illustrating the extent to which terrain-related adaptations were consistently observed across individuals, while also highlighting natural inter-individual variability in baseline gaze and locomotor behaviour.

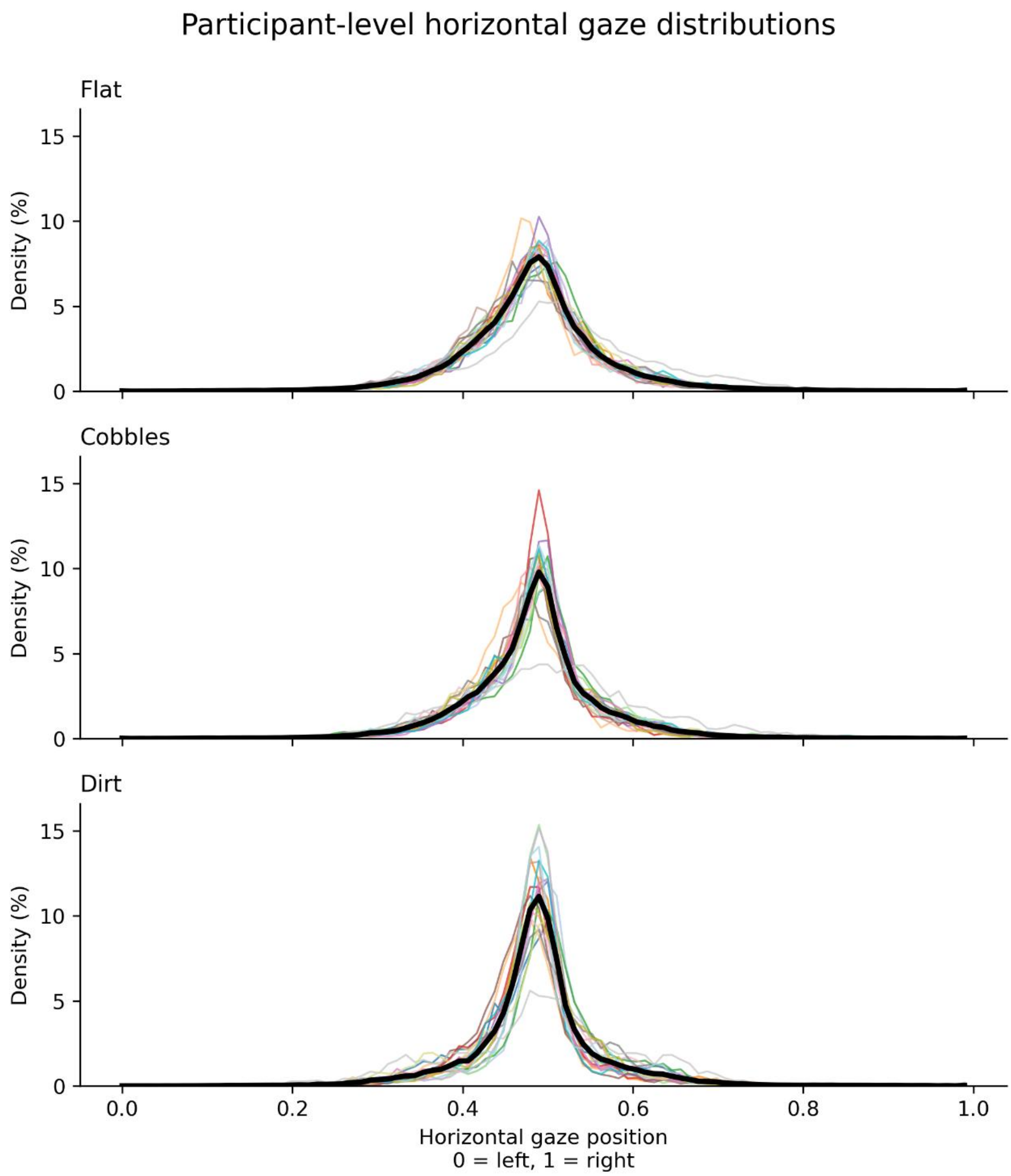

Supplementary Figure S1. Participant-level horizontal gaze distributions during walking on flat pavements, cobblestones, and dirt paths. Each coloured line corresponds to an individual participant, while the thick black line represents the group mean distribution. The three terrain conditions are

presented as vertically stacked panels with identical y-axis scaling to facilitate visual comparison across conditions.

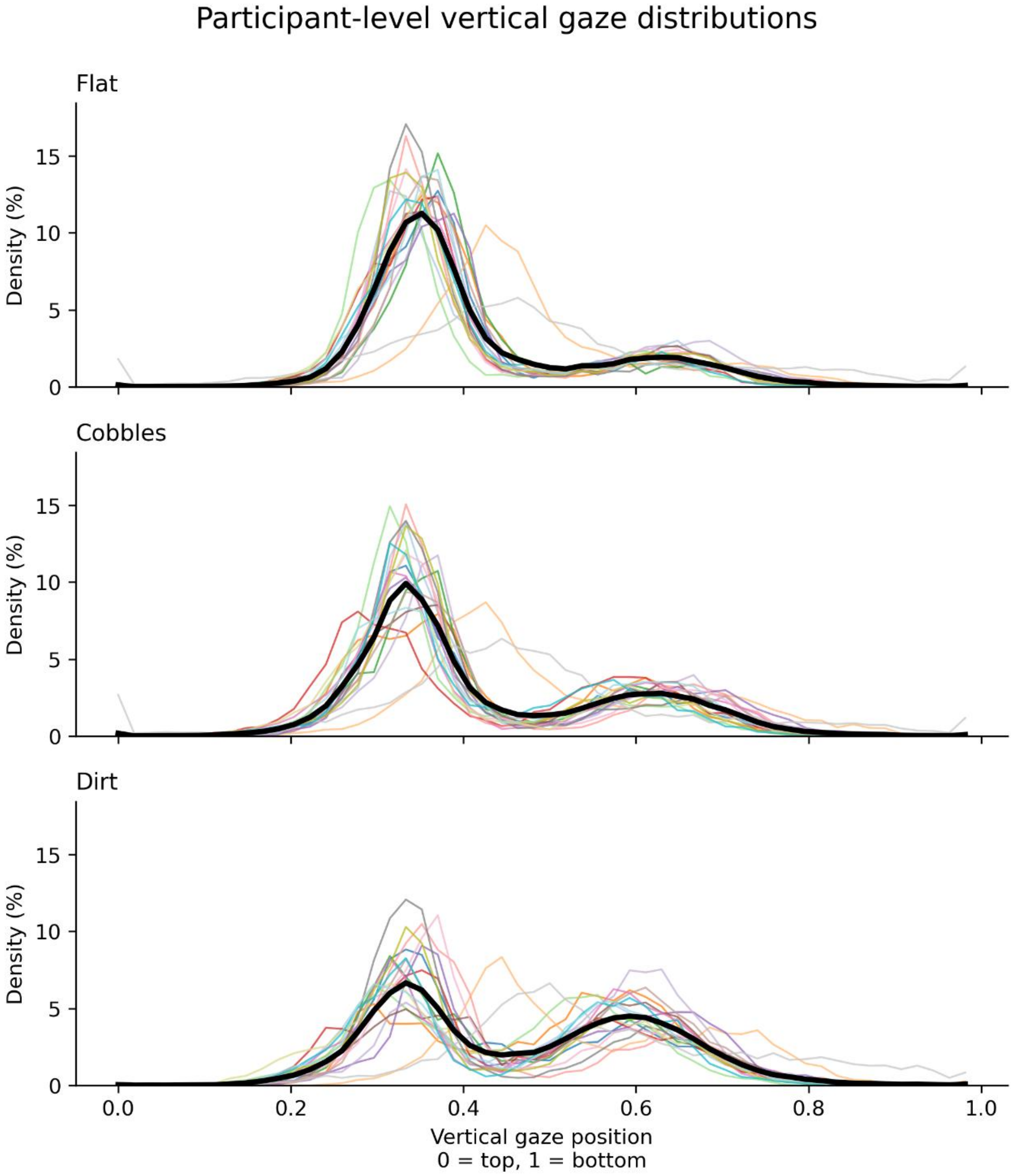

Supplementary Figure S2. Participant-level vertical gaze distributions during walking on flat pavements, cobblestones, and dirt paths. Each coloured line corresponds to an individual participant, while the thick black line represents the group mean distribution. The three terrain conditions are presented as vertically stacked panels with identical y-axis scaling to facilitate visual comparison across conditions.

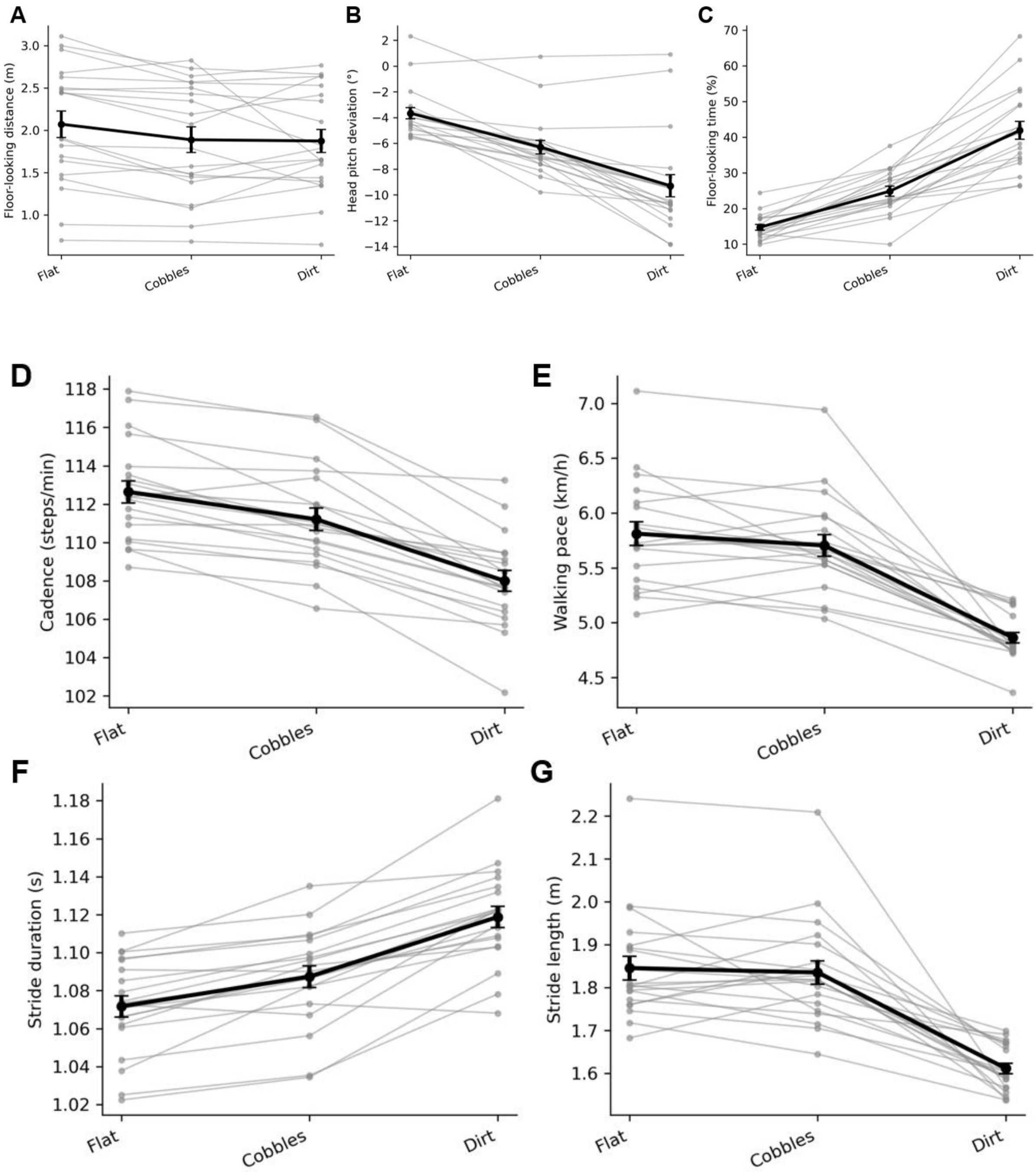

Supplementary Figure S3. Participant-level gaze, head-orientation, and gait measures across terrain types. (A) Mean floor-looking distance, (B) mean head-pitch deviation, (C) proportion of time spent fixating the floor, (D) cadence, (E) walking pace, (F) stride length, and (G) stride duration across terrain conditions. Grey lines represent individual participants, illustrating within-subject changes across flat pavements, cobblestones, and dirt paths. Thick black lines and error bars indicate the group mean ± SEM.
